# Disabled-1 Alternative Splicing in Human Fetal Retina and Neural Tumors

**DOI:** 10.1371/journal.pone.0028579

**Published:** 2011-12-06

**Authors:** Sachin Katyal, Darryl D. Glubrecht, Lei Li, Zhihua Gao, Roseline Godbout

**Affiliations:** Department of Oncology, Cross Cancer Institute, University of Alberta, Edmonton, Alberta, Canada; Dalhousie University, Canada

## Abstract

**Background:**

The Reelin-Dab1 signaling pathway plays a critical role in the positioning of migrating neurons, dendrite formation and lamination in the developing central nervous system. We have previously identified two alternatively spliced forms of Dab1 in the developing chick retina: an early form, Dab1-E, expressed in retinal progenitor cells, and a late form, Dab1 or Dab1-L, expressed in amacrine and ganglion cells. Compared to Dab1-L, Dab1-E lacks two exons that encode two Src family kinase (SFK) phosphorylation sites.

**Principal Findings:**

Both Dab1-L and Dab1-E-like transcripts were identified in human fetal retina. Expression of human Dab1-L in primary chick retinal cultures resulted in Reelin-mediated induction of SFK phosphorylation and formation of neurite-like processes. In contrast, human Dab1-E-expressing cells retained an undifferentiated morphology. The human *Dab1* gene is located within a common fragile site, and it has been postulated that it may function as a tumor suppressor. Analysis of Dab1 splice forms in retinoblastoma and neuroblastoma tumor cells revealed relative enrichment of Dab1-L-like (includes exons 7 and 8) and Dab1-E-like (excludes exons 7 and 8) transcripts in retinoblastoma and neuroblastoma, respectively. Treatment of retinoblastoma cell line RB522A with Reelin resulted in increased tyrosine phosphorylation of Dab1. As Nova2 has previously been implicated in the exclusion of exons 9B and 9C in Dab1, we examined the expression of this splicing factor in neuroblastoma and retinoblastoma cell lines. Nova2 was only detected in neuroblastoma cells, suggesting a correlation between Nova2 expression and increased levels of Dab1-E-like splice forms in neuroblastoma.

**Conclusions:**

These results indicate that alternative splicing of Dab1 is conserved in avian and mammalian species, with Dab1-L driving SFK phosphorylation in both species. Dab1-E- and Dab-L-like isoforms are also expressed in childhood neural tumors, with preferential enrichment of Dab1-L-like and Dab1-E-like isoforms in retinoblastoma and neuroblastoma, respectively.

## Introduction

Disabled-1 (Dab1) is a cytoplasmic adaptor protein that is phosphorylated when the secreted extracellular matrix glycoprotein Reelin binds to cell surface receptors apolipoprotein E receptor 2 (ApoER2) and very low density lipoprotein receptor (VLDLR) [1], [2], [3], [4], [5]. Binding of Reelin to its receptors and the ensuing Dab1 phosphorylation stimulates Src family kinases (SFK) which in turn enhances Dab1 phosphorylation [6], [7], [8], [9]. Well-defined roles for the Reelin-Dab1 signaling pathway include proper positioning of migrating neurons and dendrite formation in the central nervous system (CNS) (rev. in [10]). In mice, inactivation of Reelin, Dab1, or a combination of the two Reelin receptors, ApoER2 and VLDLR, results in inversion of neuronal layers in the cerebral cortex, laminar defects in the cerebellum and hippocampus, as well as altered dendrite formation [11], [12], [13], [14].

Like brain, the retina is a highly organized laminated structure characterized by migration of neuronal cells, positioning of neuronal cells into specific layers, outgrowth of dendrites and axons, and intercellular communication through synaptic circuitry. There are six classes of neuronal cells in the retina (ganglion, amacrine, bipolar, horizontal, cone and rod photoreceptors), located in the three nuclear layers (ganglion, inner and outer) separated by inner and outer plexiform layers. Structural analysis of the retina in Reelin and Dab1-deficient mice reveals a number of abnormalities including reduced density of amacrine dendrites, and alteration in the layering of amacrine cell processes in the inner plexiform layer [15], [16].

We have discovered a developmentally-regulated alternatively-spliced form of Dab1 called Dab1-E (early) which is specifically expressed in retinal progenitor cells of the chick embryo [17]. In contrast, the well-characterized late form of Dab1 (Dab1-L) is expressed in amacrine and ganglion cells. A key difference between the early and late forms of Dab1 is the exclusion in Dab1-E of two exons containing two SFK tyrosine phosphorylation sites (Y^185^QTI, Y^198^QY^200^I) implicated in Reelin-Dab1 signaling [18]. Of note, splicing out of the two exons results in the formation/retention of two Abl/Crk recognition sites in Dab1-E (Y^185^QVP, Y^232^DVP) [2], [17], [19].

Transfection of a GFP-tagged Dab1-L expression construct into primary chick retinal cultures results in the formation of numerous neurite-like processes, increased levels of phosphotyrosine and SFK activation [17]. None of these changes are observed upon transfection of either GFP-tagged Dab1-E or GFP control expression constructs. Mutation analysis of the tyrosine phosphorylation sites in chicken Dab1-L indicates an essential role for SFK phosphorylation site Y198, with all four tyrosine phosphorylation sites required for maximal Dab1 phosphorylation, SFK activation and neurite formation [17], [20]. These results are in general agreement with previous results in mice and tissue culture [5], [18], [21]. In contrast to Dab1-L which is phosphorylated at tyrosine residues, Dab1-E does not appear to be phosphorylated at the two remaining tyrosine residues but is phosphorylated at multiple serine/threonine residues [22].

Dab1-E-like isoforms have been reported in chicken, pig, mice and zebrafish, suggesting a widespread role for Dab1-E in vertebrates [17], [23], [24], [25]. The presence of this Dab1 isoform during development has important implications to our understanding of the Reelin-Dab1 pathway. For example, expression of Dab1-E in undifferentiated neuroblasts may serve as an effective and versatile means of ensuring that the Reelin-Dab1 signaling pathway is not prematurely activated in neuronal progenitor cells during retina and brain development. Knock-down of Dab1-E in developing chick retina indicates a role for this Dab1 isoform in the maintenance of the retinal progenitor pool [26]. In the present study, we examine the expression of Dab1 isoforms in human fetal retina, and study the role of human Dab1-E and Dab1-L using primary retinal cultures. As the *Dab1* gene is located within an unstable region of the human genome and has been proposed to function as a tumor suppressor, we also investigate Dab1 expression and splicing in retinoblastoma and neuroblastoma cells.

## Results

### Dab1 is alternatively spliced in human fetal retina and brain

**Figure 1:**
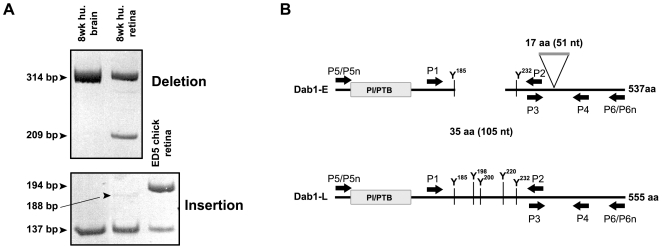
RT-PCR analysis of Dab1 deletion and insertion regions. (**A**) cDNAs synthesized from poly(A)^+^ RNA from human fetal brain (8 wks gestation), retina (8 wks gestation) and chick retina (E5) were amplified using P1 and P2 primers for deletion analysis and P3 and P4 primers for insertion analysis. Sizes of amplified bands are indicated. (**B**) Schematic representation of human Dab1-E and Dab1-L proteins and relative positions of primers used for RT-PCR amplification.

Dab1 is alternatively spliced as a function of developmental stage in chick retina resulting in the formation of two main isoforms: Dab1-E expressed in retinal progenitor cells and Dab1-L expressed in amacrine and ganglion cells [17]. In comparison to the well-characterized Dab1-L isoform, Dab1-E lacks two exons containing two SFK-mediated tyrosine phosphorylation sites and appears to function independently of SFK phosphorylation [22], [26]. Dab1-E also contains an extra 57 bp exon that is not present in Dab1-L. We used primers flanking the two-exon deletion region (P1, P2) and the insertion region (P3, P4) to determine whether similar alternative splicing events also occur in human fetal retina. RT-PCR analysis of human fetal retina at 8 wks gestation using P1 and P2 primers generated two bands, of 314 bp and 209 bp, with the latter spanning the 105 bp two-exon (exons 7 and 8) deletion region (Figs. 1A, B). These two bands are of the same sizes as those obtained with E5 chick retina cDNA. Similar results were obtained with human fetal brain at 8 wks gestation, except that the 209 bp band was barely detectable. The inclusion and exclusion of exons 7 and 8 in the 314 bp and 209 bp bands, respectively, was verified by sequencing.

**Figure 2:**
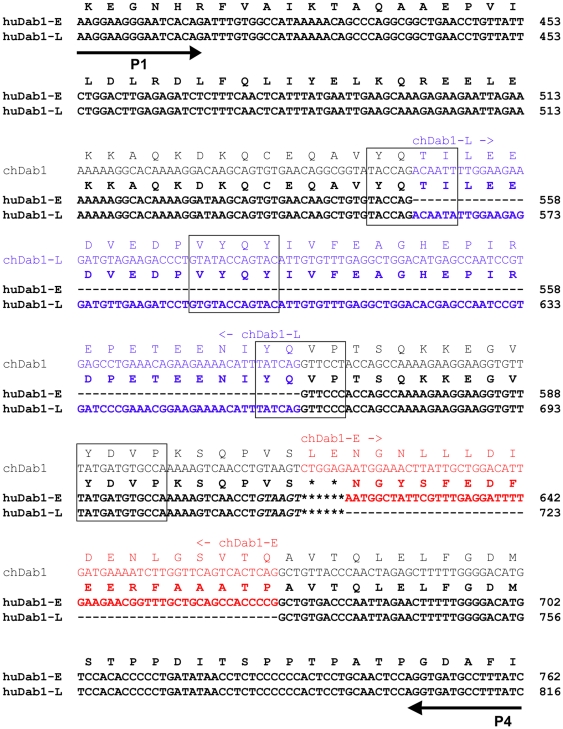
Sequence alignment of P1/P4-amplified Dab1 fragments. HuDab1-E, huDab1-L and chDab1 nucleotide and amino acid sequences are as indicated. The Dab1-L-specific 105 nt two-exon (7/8) region is indicated in blue while the huDab1-E-specific 51 nt exon 9B (57 nt in the case of chDab1-E) is indicated in red. SFK (Y185 and Y198) and Abl/Crk (Y220 and Y232) recognition motifs are boxed. The six italicized nucleotides were only present in a subset of the products analysed.

RT-PCR analysis of human fetal retina using P3 and P4 primers generated two bands, of 188 bp and 137 bp, with the former predicted to encompass the insertion exon unique to Dab1-E. While clearly visible, the 188 bp band was considerably weaker than the 137 bp band. The 188 bp band was not detected in 8 wk human fetal brain. Sequence analysis of the 188 bp and 137 bp bands revealed an extra 51 bp in the top band, corresponding to the 57 bp exon previously identified in chick Dab1-E [17]. Based on the published exon/intron structure of the human *Dab1* gene, this 51 bp exon corresponds to exon 9-2 (labeled 9B in this manuscript), and has previously been shown to be alternatively spliced in embryonic mouse brain and chick eye [23]. The amino acid sequence of the two-exon deletion region (exons 7 and 8), the 51 bp insertion region (exon 9B) and sequence alignment of the region amplified in huDab1-E and huDab1-L by the P1/P4 primer set are shown in Fig. 2.

### Human Dab1-L promotes neurite formation and SFK activiation

**Figure 3:**
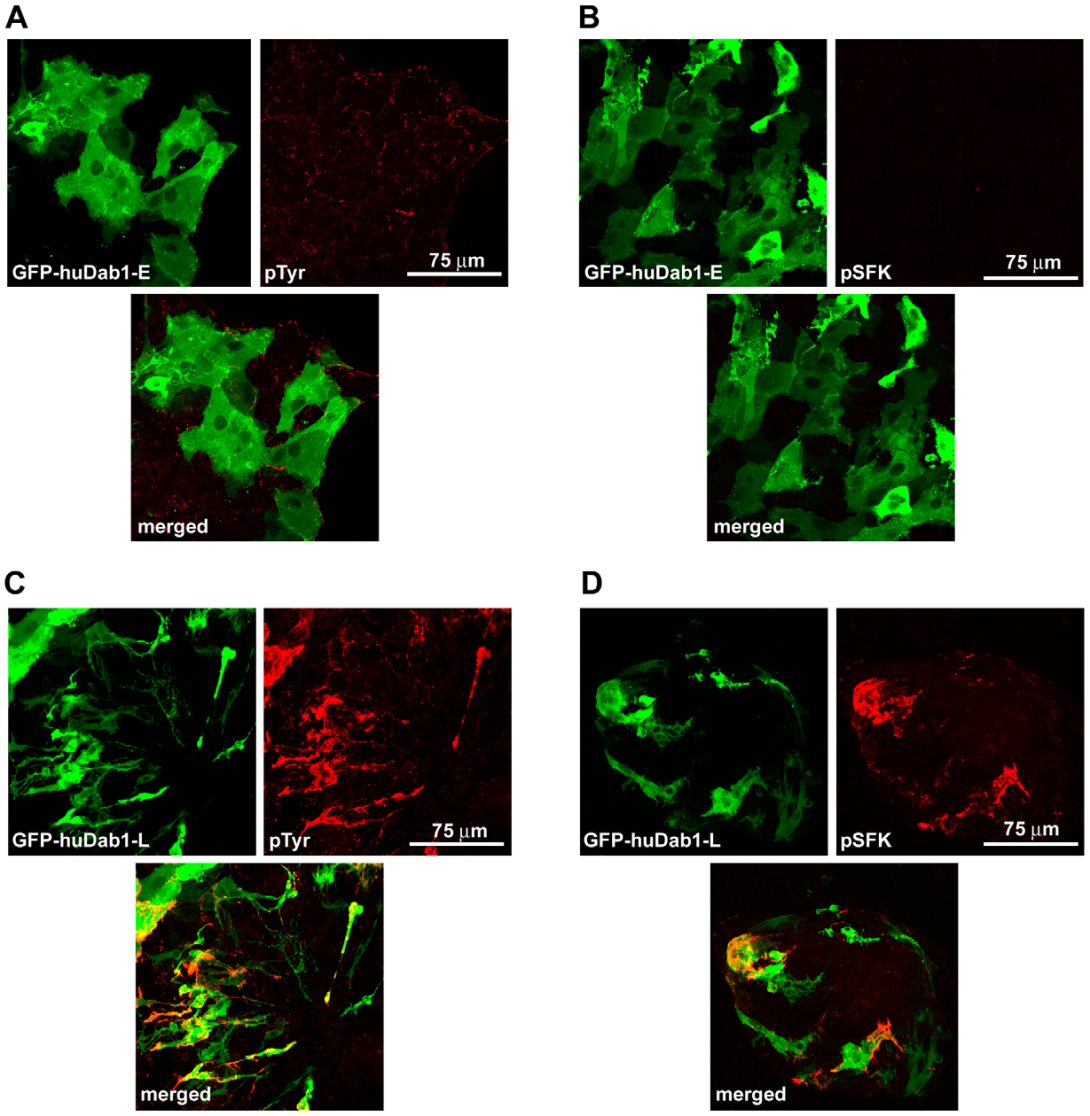
Analysis of Dab1-transfected primary chick retinal cultures. (**A** and **C**) GFP expression and phosphotyrosine (pTyr) immunoreactivity in GFP-huDab1- (E and L) transfected retinal cultures. (**B** and **D**) GFP and phosphoSFK^Y416^ (pSFK) expression in primary retinal cultures transfected with 2X CsCl-purified GFP-huDab1-E and -L expression constructs. Cells were fixed with 4% paraformaldehyde, permeabilized and stained with either anti-phosphotyrosine or anti-phosphoSFK^Y416^ antibodies followed by Alexa 555-conjugated goat anti-mouse secondary antibody. The GFP signal in transfected cells was detected by epifluorescence. Similar results were obtained in three independent experiments.

To isolate full-length human Dab1-E and Dab1-L (huDab1-E and huDab1-L), human fetal retina cDNA was amplified using nested primers P5/P6 and P5n/P6n, with the latter containing *Eco*RI and *Bam*HI restriction enzyme sites, respectively. Of the 12 clones analysed, one corresponded to huDab1-E and eleven to huDab1-L. Full-length huDab1-E and huDab1-L cDNAs were verified by sequencing and cloned into pEGFP-C1. We then transfected GFP-tagged huDab1-E and huDab1-L expression constructs into primary chick retina cultures. These cultures have previously been shown to express Reelin [20]. Cells which expressed human Dab1-L formed numerous neurite-like processes (Figs. 3C, D), similar to those observed upon transfection of chicken Dab1-L [17]. In contrast, huDab1-E-expressing cells retained an undifferentiated epithelial-like appearance, similar to that obtained with GFP-transfected cells (Figs. 3A, B; data not shown). Dab1-L-expressing cells also showed increased tyrosine phosphorylation (compare Fig. 3C with 3A) and SFK activation (compare Fig. 3D with 3B). These data suggest that Dab1 functions associated with SFK activation are conserved in human and chicken.

**Figure 4:**
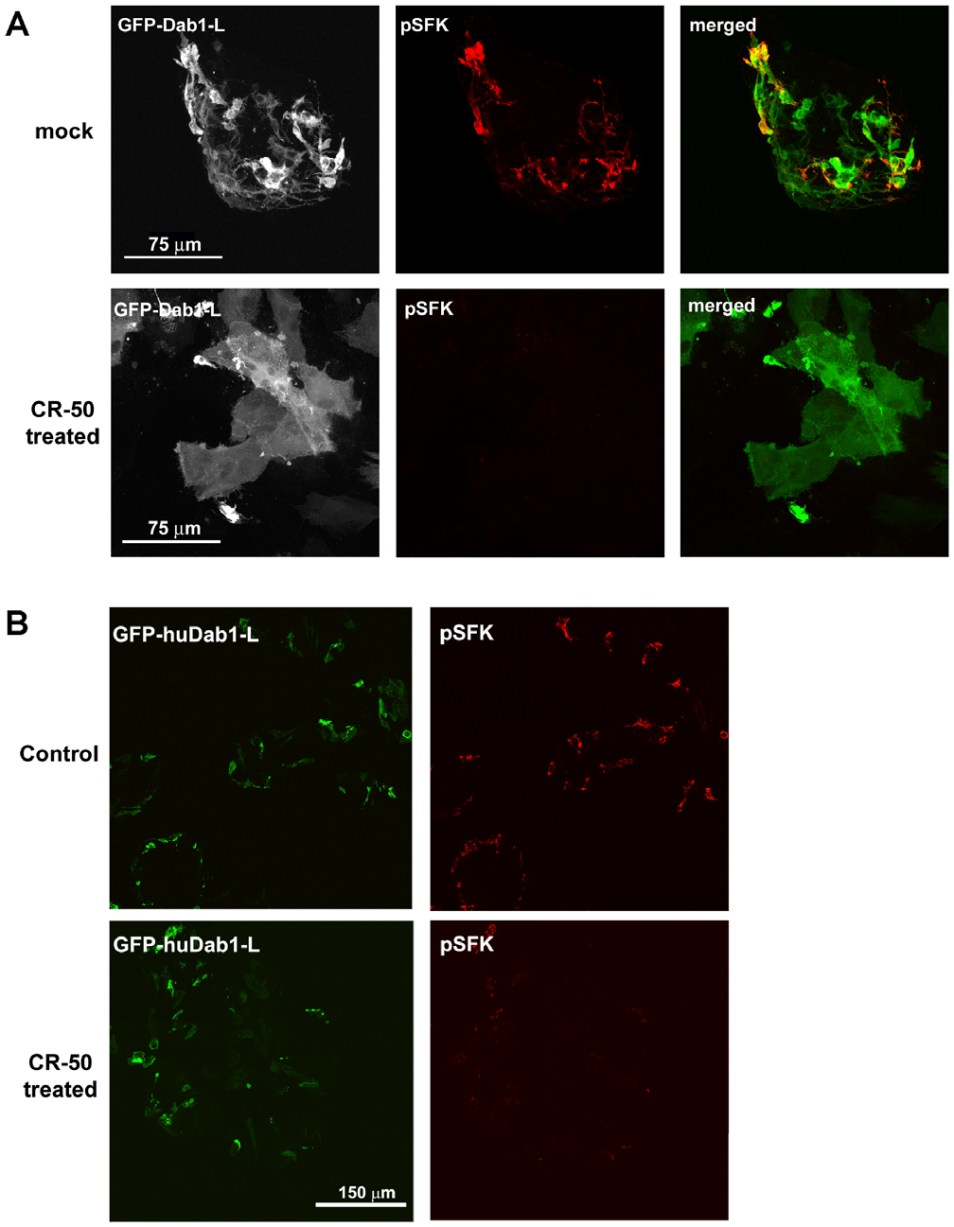
Analysis of CR-50-treated huDab1-L-expressing chick retinal cells. GFP-huDab1-L and phospho-SFK^416^ (pSFK) expression in mock and CR-50-treated cells. Sixteen hours after DNA transfection, cells were washed and exposed to two sequential treatments of 1 µl/ml CR-50 at 24 h per treatment. Cells were fixed with 4% paraformaldehyde, permeabilized and immunostained with anti-phosphoSFK^Y416^ antibody followed by Alexa 555-conjugated goat anti-mouse secondary antibody. The GFP signal in transfected cells was detected by epifluorescence. (**A**) High magnification view of transfected cells. (**B**) Low magnification view of transfected cells using the Tile-scan function of the LSM program. A 3×3 tile was used to construct each image. Similar results were obtained in two different experiments.

To determine whether Dab1-L-mediated SFK activation and associated morphological changes are Reelin-dependent, we treated GFP-huDab1-L transfected retinal cells with an anti-Reelin antibody, CR-50, previously shown to prevent Reelin dimerization and binding to receptors thereby abrogating Reelin-mediated signaling [1], [27]. Dab1-L-expressing cells treated with CR-50 for two days showed a dramatic reduction in SFK activity compared to mock-treated Dab1-L-expressing cells (Figs. 4A, B). Furthermore, cells treated with CR-50 had a round, undifferentiated morphology similar to that of Dab1-E-expressing cells. These data indicate that Reelin signaling is required for Dab1-L function in retinal cultures.

### Dab1 expression correlates with SFK activation in human fetal retina tissue

**Figure 5:**
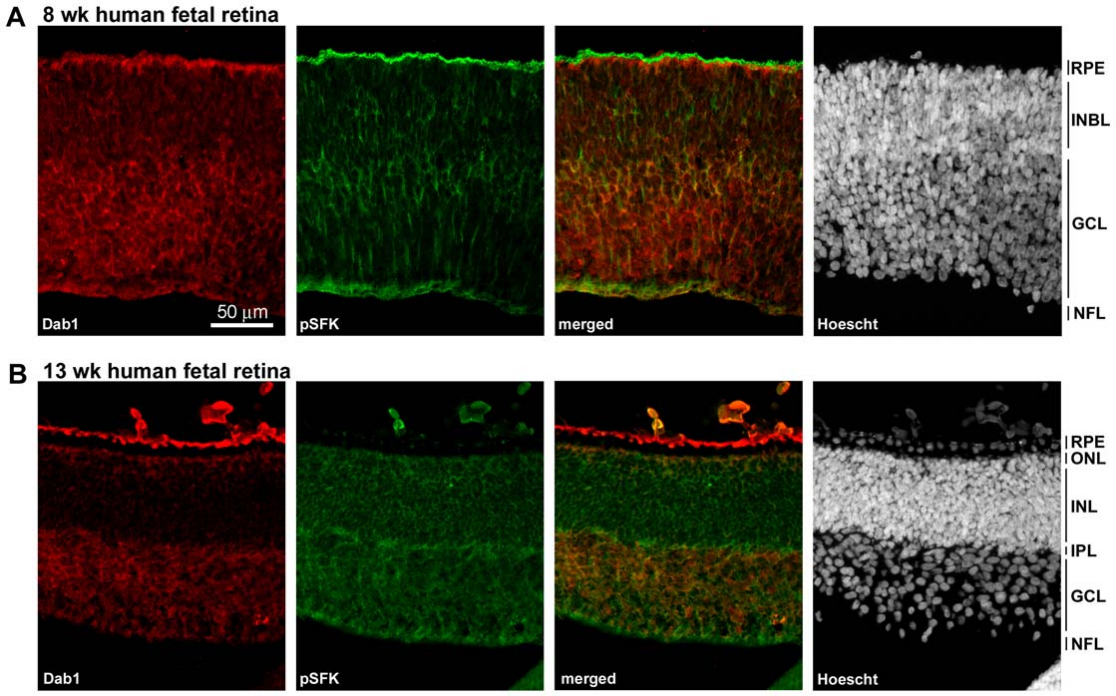
Immunofluorescence analysis of Dab1 (red) and phospho-SFK^Y416^ (green) in the developing human retina. Sections were double-stained with anti-Dab1 and anti-phospho-SFK^Y416^ (pSFK) antibodies followed by Alexa 555-conjugated goat anti-rabbit and Alexa 488-conjugated donkey anti-mouse secondary antibodies. Sections were counterstained with the fluorescent dye Hoescht 33258 to label the nuclei. Retinal tissue sections were prepared from human fetal retina at 8 wks gestation (**A**) and 13 wks gestation (**B**). The strong staining observed in the RPE is caused by autofluorescence. Abbreviations are: RPE, retinal pigment epithelium; INBL, inner neuroblastic layer; NFL, nerve fiber layer; ONL, outer nuclear layer; OPL, outer plexiform layer; INL, inner nuclear layer; IPL, inner plexiform layer; GCL, ganglion cell layer.

Frozen human fetal retina tissue sections at 8 and 13 wks gestation were double-stained with anti-Dab1 (red) and anti-phospho-SFK^Y416^ antibodies (green). Analysis of the 8-wk retina revealed weak Dab1 immunostaining in the undifferentiated inner neuroblastic layer (INBL) and stronger staining of the ganglion cell layer (GCL) (Fig. 5A). The pattern for activated SFK (Fig. 5A) was similar, with highest levels in the GCL and ganglion nerve fiber layer (NFL). By 13 wks gestation, the Dab1 signal was much more pronounced in the GCL compared to the inner nuclear layer (INL), although the emerging outer nuclear layer (ONL) was also positive (Fig. 5B). Dab1-expressing ganglion cells were positive for activated SFKs (Fig. 5B).

**Figure 6:**
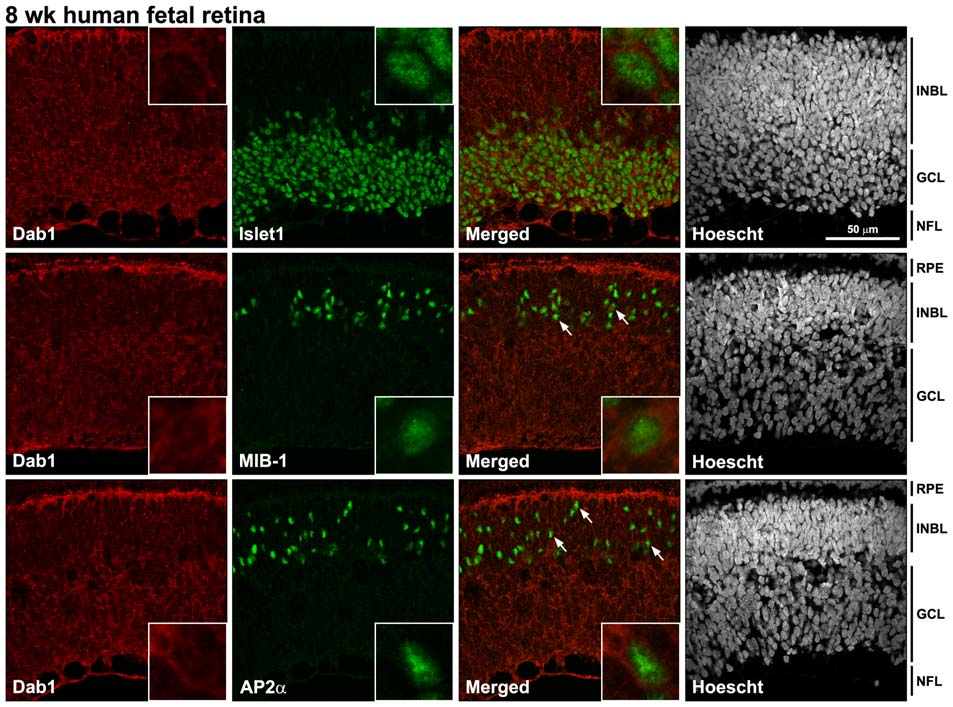
Co-staining of Dab1 and specific retinal lineage markers at 8 wks gestation. Retinal tissue sections were double-stained with antibodies to Dab1 and Islet1 (marker of ganglion cells), Dab1 and MIB-1 (proliferation marker), and Dab1 and AP2α (marker of amacrine cells). Cells that are co-stained with anti-Dab1 and MIB1, and anti-Dab1 and AP2α are indicated by the arrows. Insets show higher magnification of double-stained cells. Abbreviations are: RPE, retinal pigment epithelium; INBL, inner neuroblastic layer; NFL, nerve fiber layer; GCL, ganglion cell layer.

**Figure 7:**
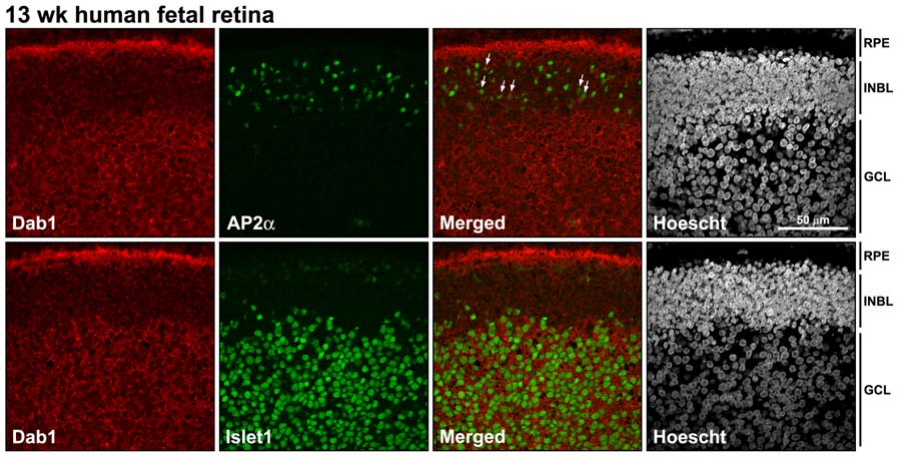
Co-staining of Dab1 and specific retinal lineage markers at 13 wks gestation. Retinal tissue sections were double-stained with antibodies to Dab1 and Islet1 (marker of ganglion cells), and Dab1 and AP2α (marker of amacrine cells). Cells that are co-stained with anti-Dab1 and AP2α are indicated by the arrows. Abbreviations are: RPE, retinal pigment epithelium; INL, inner nuclear layer; GCL, ganglion cell layer.

Next, we co-immunostained 8 wk human fetal retina tissue sections with antibodies to Dab1 and Islet1 (marker of ganglion cells), Dab1 and MIB-1 (marker of proliferating cells), and Dab1 and AP2α (marker of amacrine cells) (Fig. 6). Dab1 was expressed throughout the ganglion cell layer, with co-staining also observed in some proliferating and amacrine cells (indicated by arrows and insets in Fig. 6). Co-immunostaining of 13 wk fetal retina tissue sections with antibodies to Dab1 and Islet1 revealed widespread expression of Dab1 in ganglion cells (Fig. 7). Dab1 staining was also observed in amacrine cells (indicated by arrows in Fig. 7). These results are consistent with huDab1-E expression in proliferating cells and huDab1-L expression in ganglion and amacrine cells, as previously described in chick retina [17], [26].

### Retinoblastoma and neuroblastoma tumor cells produce multiple alternatively-spliced Dab1 transcripts

**Figure 8:**
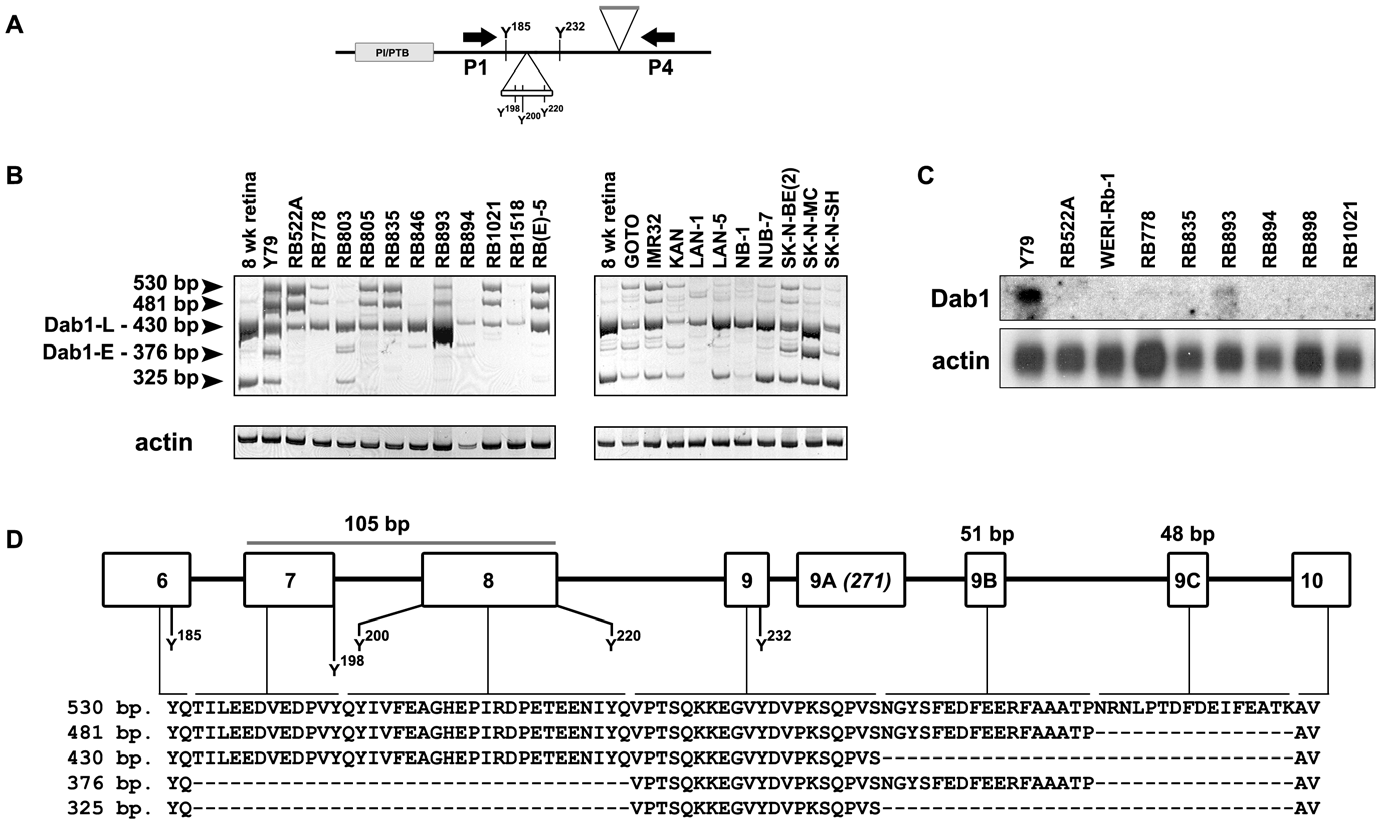
Identification of *Dab1* splice forms expressed in RB and NB cell lines. (**A**) Schematic representation of Dab1 protein showing the region encompassing exons 7 and 8 which contains SFK and Abl/Crk recognition motifs and exons 9, 9B and 9C. The P1 and P4 primers used for the RT-PCR analysis span the entire exon exclusion/inclusion region. (**B**) DNA amplified in RB cell lines (left panel) and NB cell lines (right panel) using primer set P1/P4. Sizes of amplified DNAs are as indicated and sequences are shown in **D**. Amplification of actin cDNA was used as a control to ensure that similar amounts of cDNA were used for each cell line. (**C**) Northern blot analysis of *Dab1* in RB cell lines. Actin served as the loading control. (**D**) Alignment of Dab1 sequences corresponding to *Dab1* splice forms expressed in RB and NB cell lines using primer set P1/P4. The human *Dab1* genomic intron/exon structure spanning exon 6 to exon 10 is schematically represented. The amino acid sequence encoded by the 530, 481, 430, 376 and 325 bp DNA fragments amplified in **B** are aligned to show exon inclusion/exclusion in each *Dab1* splice form.

RB tumors have been postulated to be derived from a neuronal precursor cell committed to the amacrine and/or horizontal lineage [28], [29], [30], [31], [32]. NB tumors are believed to be derived from neuroectodermal/neural crest cells [33]. To investigate whether either Dab1-E or -L is expressed in RB and NB tumors, we carried out RT-PCR analysis of 12 RB tumor cell lines and 10 NB cell lines. Dab1 products were observed in all RB and NB tumor cell lines analysed, although there were significant variations in relative band intensities in the different cell lines (Fig. 8). Using the P1 and P4 primers to amplify the entire region spanning the insertion/deletion region (Fig. 8A), we observed five major products, all of which are clearly depicted in the Y79 (RB) lane (Fig. 8B). Based on sequence analysis: (i) the 530 bp band includes exons 7, 8, 9, 9B and 9C, (ii) the 481 bp band includes exons 7, 8, 9 and 9B, (iii) the 430 bp band, corresponding to Dab1-L, includes exons 7, 8 and 9, (iv) the 376 bp band, corresponding to Dab1-E, includes exons 9 and 9B, and (v) the 325 bp band includes only exon 9 (Fig. 8D). Of note, the 430 bp band, representing Dab1-L, was present in relatively high abundance in the majority of RB and NB cell lines. Higher molecular weight products containing exons 7/8/9/9B or exons 7/8/9/9B/9C were predominant in RB lines, whereas lower molecular products excluding exons 7/8/9B or exons 7/8/9B/9C were predominantly found in NB cell lines. These results suggest that splicing is controlled differently in RB and NB cells, perhaps reflecting the nature of the *trans*-acting splicing factors expressed in these two tumors. Of note, the 325 bp DNA fragment which excludes exons 7/8/9B/9C, was more abundant in human fetal retina than the 376 bp DNA fragment which corresponds to Dab1-E. The counterpart of the 325 bp DNA band was not observed in chick retina at any of the developmental stages tested, suggesting that this may represent an intermediate splice product or a final splice product specific to human Dab1.

**Figure 9:**
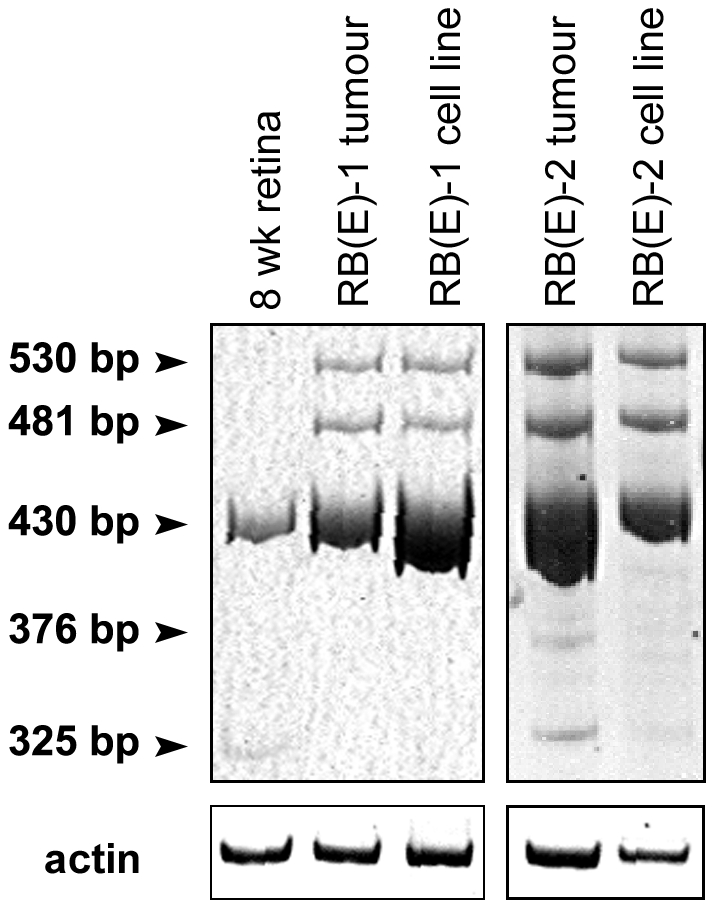
Comparison of *Dab1* splice forms expressed in two primary RB tumors and corresponding cell lines. cDNAs derived from RB tumors and cell lines were amplified using primer set P1/P4. The sizes of the amplified bands are as indicated.

To address whether alternative splicing of Dab1 might be affected by growth of tumor cells in culture, RT-PCR analysis with the P1/P4 primer set was carried out using total RNA isolated from two RB tumor biopsies. The corresponding cell lines were included for comparison. A remarkable level of similarity, both at the quantitative and qualitative levels, was observed between matching tumors and cell lines. As previously noted, the 430 bp band, representing Dab1-L, was predominant in both the tumors and their corresponding cell lines (Fig. 9). The 481 bp and 530 bp bands were also present in both sets of tumors and cell lines. These data indicate that Dab1 splicing and Dab1 function in RB cells have not been significantly altered as the result of growth in tissue culture, in keeping with the observation that RB cell lines appear stable in culture, with few karyotypic and morphological changes [34], [35]. A similar analysis could not be carried out with NB tumors as no fresh tumor material with corresponding cell line was available.

We have also carried out northern blotting of 9 RB cell lines. *Dab1* transcripts were either undetectable or weakly expressed in the majority of these cell lines (Fig. 8C). Highest levels of *Dab1* RNA were observed in Y79 followed by RB893, consistent with RT-PCR data.

### Analysis of Reelin and Dab1 protein levels in RB and NB cell lines

**Figure 10:**
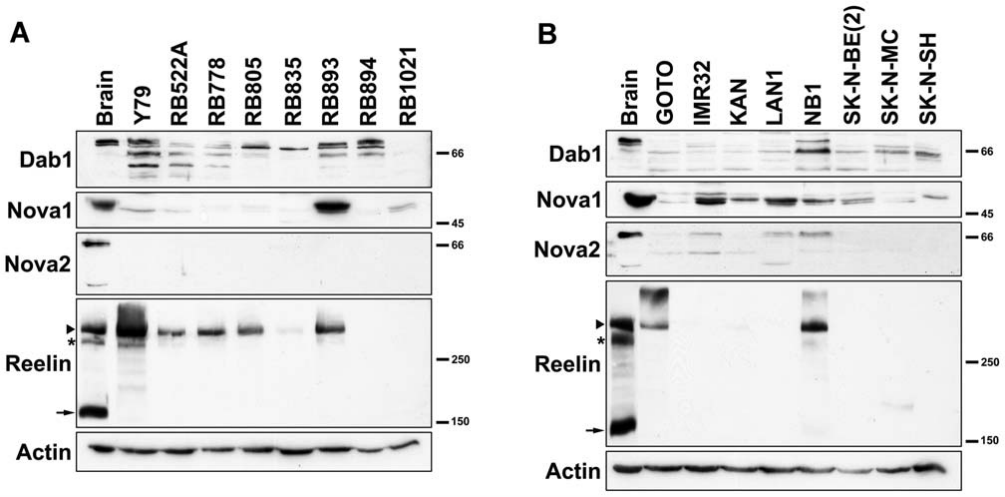
Western blot analysis of RB (A) and NB (B) cell lines. Fifty µg of protein lysates from each of the indicated tumor cell lines were electrophoresed through a SDS- polyacrylamide gel (10% polyacrylamide for Dab1, actin, Nova1, Nova2; 8% (low BIS) for Reelin) and electroblotted onto nitrocellulose or PVDF membranes. Blots were immunostained with rabbit anti-Dab1 (1∶2000), rabbit anti-Nova1 (1∶1000), goat anti-Nova2 (1∶1500), mouse anti-Reelin (1∶500) or mouse anti-actin (1∶300,000) antibodies, followed by labeling with appropriate HRP-conjugated secondary antibodies. Arrowhead, asterisk and arrow point to full-length Reelin (3428 amino acids), N-R6 Reelin (N-terminal to end of repeat 6) and N-R2 Reelin (N-terminal to end of repeat 2), respectively [48].

To address whether the Reelin-Dab1 signaling pathway is active in RB and NB cell lines, we prepared whole cell lysates from eight RB, seven NB cell lines, with human fetal brain as control tissue. Western blot analysis of these extracts identified a number of bands in RB cell lines using anti-Dab1 antibody (Fig. 10). These bands likely represent different isoforms of Dab1 as well as post-translationally-modified forms of Dab1. In general, the intensity of the Dab1 protein bands was weaker in NB compared to RB cell lines, and Dab1 bands in NB cells had a faster migration rate. Full-length Reelin was expressed in the majority of RB cell lines tested (Y79, RB522A, RB778, RB805, RB835, RB893), with N-terminally truncated Reelin also detected in Y79 (Fig. 10). Two NB cell lines (GOTO and NB1) expressed full-length Reelin as well as a higher molecular weight form of Reelin. None of the RB and NB cell lines expressed the 180 kDa cleaved (activated) form of Reelin [10].

**Figure 11:**
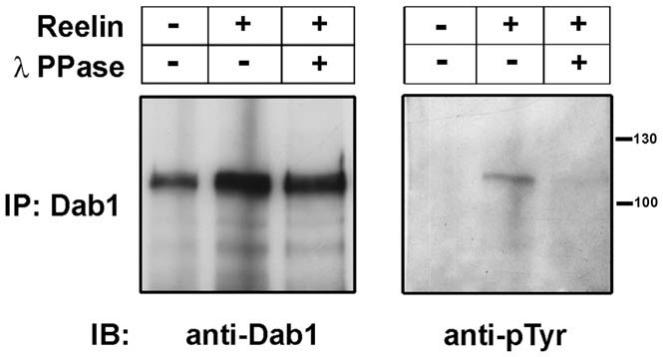
Induction of Dab1 tyrosine phosphorylation in Reelin-treated RB522A cells. Dab1 was immunoprecipitated from mock-treated and Reelin-treated RB522A cells. To examine the phosphorylation status of Dab1, immunoprecipitates were further digested with lambda phosphatase. Immunoprecipitates were electrophoresed in an 8% SDS-polyacrylamide gel and immunoblotted with anti-phosphotyrosine antibody (4G10), followed by anti-Dab1 antibody.

To investigate whether exogenous Reelin can induce Dab1 tyrosine phosphorylation in tumor cells, we treated RB522A cells with Reelin. Cells were harvested 30 minutes after addition of Reelin and lysates prepared in the presence of protease and phosphatase inhibitors. Dab1 was immunoprecipitated with anti-Dab1 antibody and western blot analysis carried out with 4G10, an antibody that specifically recognizes phosphorylated tyrosine residues. Although the signal intensity was weak, we observed increased levels of tyrosine-phosphorylated Dab1 in cells treated with Reelin compared to mock-treated cells (Fig. 11). Lambda phosphatase-digestion of immunoprecipitated Dab1 resulted in a decrease in levels of tyrosine-phosphorylated Dab1 (Fig. 11).

Nova splicing factors have been implicated in the regulation of Dab1 alternative splicing [36], [37]. In particular, *Nova2* knock-out mice show increased levels of Dab1 isoforms that include exons 9B and 9C [36]. We therefore investigated whether Nova2 and family member Nova1 are expressed in RB and NB cell lines. As shown in Fig. 10, Nova1 was prominent in most of the RB and NB cell lines tested. In contrast, Nova2 was detected in a subset of NB cell lines, but not in any of the RB cell lines tested.

## Discussion

In this study we demonstrate that the two alternatively-spliced forms of Dab1, Dab1-E and Dab1-L, previously identified in embryonic chick retina, are also present in human fetal retina. Alternative splicing results in the exclusion of two exons containing two SFK recognition motifs in huDab1-E, whereas two Abl/Crk recognition motifs are retained in both huDab1 splice forms. One of these SFK motifs encompasses tyrosine-198, an essential residue for Reelin-induced tyrosine phosphorylation of Dab1 [18]. *Dab1* alternative splicing also results in the inclusion of a 51 nt exon (exon 9B) of unknown function in *huDab1-E* which corresponds to the 57 nt exon in *chDab1-E*. Transfection of a GFP-tagged huDab1-L expression construct into primary chick retinal cultures has the same effect as chDab1-L transfection; i.e. formation of neurite-like processes, increased phosphotyrosine levels and activation of SFKs. None of these morphological and biochemical features are observed when GFP-huDab1-L-transfected cells are cultured in the presence of the CR-50 antibody which has been previously shown to abolish Reelin signaling [1], [27]. Chick retina cells transfected with either GFP-chDab1-E or GFP-huDab1-E expression constructs retain an undifferentiated morphology and show no increase in phosphotyrosine levels or SFK activation. These results suggest similar roles for Dab1-E and Dab1-L in vertebrate retinal development.

Exclusion and inclusion of SFK phosphorylation sites in human/chicken Dab1-E and Dab1-L, respectively, points to a novel mechanism for controlling SFK-dependent Reelin-Dab1 signaling in an environment where proliferating and differentiating cells are simultaneously exposed to secreted Reelin. Thus, the combination of extracellular Reelin and Dab1-L expression in neuronal cells ensures Reelin-Dab1 signaling through SFK, whereas Dab1-E expression in progenitor cells likely uncouples Reelin-Dab1 signaling by preventing SFK activation. Knock-down of Dab1-E at early stages of retinal development in chick embryos reduces the number of proliferating cells and promotes ganglion cell differentiation, suggesting a critical role for Dab1-E during retinal development [26].

We have previously shown that Dab1-E and Dab1-L are the two major isoforms expressed in chick [17]. In human fetal retina, a *Dab1* splice product which lacks both exons 7/8 and 9B/9C appears to be more abundant than Dab1-E which lacks exons 7/8 but retains exon 9B. This double-deleted Dab1-E-like isoform may play a role specific to human fetal retina or may represent an intermediate transcript that undergoes additional splicing events.

Based on immunostaining analysis, Dab1 is found in both the inner neuroblastic layer and ganglion cell layer of human fetal retina at 8 wks gestation. By 13 wks gestation, Dab1 is widely distributed throughout the ganglion cell layer. Amacrine cells also express Dab1 at this developmental stage. In addition, Dab1 appears to be expressed in emerging photoreceptor cells at 8 and 13 wks gestation. Dab1 has previously been reported to be expressed in the amacrine cells of newborn and adult human retina [38]. Furthermore, Dab1 has been shown to be expressed in type AII amacrine cells of mice at post-natal day 18 [15], [16]. These combined data suggest roles for Dab1 in the proliferating and ganglion cells of the developing retina, as well as in the amacrine cells of the mature retina.

The *Dab1* gene is located within an unstable region of the human genome called a common fragile site [39]. The possibility that Dab1 might function as a tumor repressor has been explored in a few studies. For example, analysis of 29 gangliogliomas by real-time RT-PCR analysis revealed reduced levels of *Dab1* RNA in tumors compared to normal brain tissue [40]. Similarly, decreased levels of Dab1 have been observed in brain and endometrial tumor tissue, as well as a variety of cancer cell lines [41]. Based on western blotting, there is little reduction in levels of Dab1 protein in RB cell lines compared to fetal brain, although there are more Dab1 isoforms in RB cells. The Reelin-Dab1 signaling pathway appears to be functional in RB cells, as Reelin treatment of RB522A cells resulted in increased levels of tyrosine-phosphorylated Dab1.

Dab1 protein was present at lower levels in NB cell lines compared to RB cell lines and fetal brain, with most of the protein bands being of a reduced size compared to fetal brain Dab1 protein. Regulation of Dab1 protein levels may be post-transcriptional as *Dab1* transcripts are present at similar levels in RB and NB cell lines based on RT-PCR analysis. *Dab1-L*-like transcripts (i.e. those that include exons 7 and 8) are more abundant in RB cell lines than *Dab1-E*-like transcripts (i.e. those that exclude exons 7 and 8). Although *Dab1-L*-like transcripts (particularly *Dab1-L*) are also prominent in NB cell lines, there is a higher abundance of *Dab1-E*-like transcripts in NB compared to RB cell lines. These results suggest that the splicing factors involved in the exclusion of exons 7 and 8 are preferentially expressed in NB compared to RB cells.

**Figure 12:**
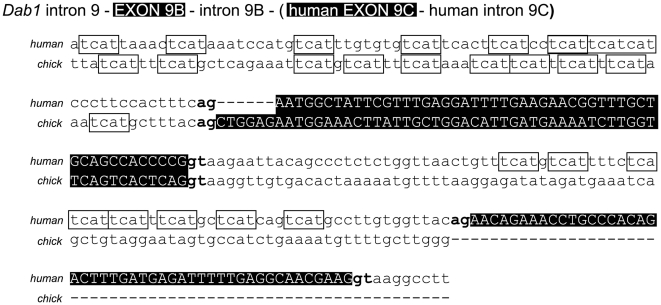
Comparison and analysis of human and chicken *Dab1* genomic DNA sequences. Sequences spanning chicken *Dab1* intron 9 to intron 9B and human *Dab1* intron 9 to intron 9C were aligned and compared. Intronic regions are in small case whereas exonic regions are shaded and in uppercase. Splice donor (gt) and acceptor (ag) sites are indicated in bold. Both human and chicken *Dab1* have an exon 9B; human *Dab1* has an additional exon 9C. TCAT sequences and corresponding Nova binding elements (UCAU in pre-mRNA) are boxed. Multiple Nova binding elements are found upstream of exon 9B (intron 9) in human and chicken *Dab1* and exon 9C (intron 9B) in human *Dab1*.

Analysis of the *Dab1* genomic sequence reveals 8-10 TCAT repeats located in human and chicken intron 9 (upstream of exon 9B). These repeats are also found in human intron 9B (upstream of exon 9C) (chicken *Dab1* genomic DNA does not contain exon 9C) (Fig. 12). The Nova family of heterogenous ribonucleoprotein (hnRNP)-like neuron-specific splicing factors binds to this element (UCAU in RNA) [42], [43]. Nova regulates alternative exon usage in a variety of genes during brain development and can function as either a splicing activator or repressor [37], [44], [45]. Of note, Nova2 has been shown to bind the UCAU clusters upstream of exons 9B and 9C (referred to as exons 7b and 7c by some investigators) in mouse *Dab1,* thus suppressing the inclusion of these two exons [36]. In this regard, it is noteworthy that Nova2 is detected in NB but not in RB cell lines, and exclusion of exons 9B and 9C (combined with exclusion of exons 7 and 8) is primarily observed in NB *Dab1* transcripts. Nova2 may therefore play a role in the exclusion of exons 9B and 9C in NB cell lines.

In summary, we report multiple Dab1 isoforms in human fetal retina, including Dab1-E and Dab1-L which have been previously reported in chick retina. Using primary retinal cultures, we show that human Dab1-L induces the formation of neurite-like processes and activates SFK in a Reelin-dependent manner. We also report that Dab1 and Reelin are expressed in RB and NB cell lines suggesting that the Reelin-Dab1 signaling pathway may be active in these tumor cells. We demonstrate that the Nova2 splicing factor is preferentially expressed in NB compared to RB cell lines, and that there is a preponderance of Dab1 products that exclude exons 7/8/9B/9C in NB compared to RB cell lines.

## Materials and Methods

### Ethics statement

Ethics approval for the collection of human fetal retina (8 weeks and 13 gestation), human fetal brain (8 weeks gestation) and human retinoblastoma was obtained from the Institutional Review Board (Alberta Cancer Board Research Ethics Committee and University of Alberta Ethics Review Committee). In the case of human fetal tissue, the need for consent was waived as the tissue was collected under a general protocol that did not require any information regarding the patient. Consent was not required at the time that the RB tumor biopsies were collected (>15 years ago). Human RB cell lines were obtained from Dr. Brenda Gallie (Ontario Cancer Institute) or were generated de novo.

### Primers

Human Dab1 P1, 5′-GGAAGGAAGGGAATCACAG-3′; human Dab1 P2, 5′-GGCACATCATAAACACCTTCC-3′; human and chick Dab1 P3, 5′-GGAAGGTGTTTATGATGTGCC-3′; human and chick Dab1 P4, 5′-ATGGGATAAAGGCATCACCT-3′; human Dab1 P5, 5′-**ATG**TCAACTGAGACAGAACTTC-3′; human Dab1 P5n, 5′-CTTCGAATTCT**ATG**TCAACTGAGACAGAACTTC-3′; human Dab1 P6, 5′-TAT*CTA*GCTACCGGCCTG-3′; human Dab1 P6n, 5′-GCAAGGATCCCTAT*CTA*GCTACCGGCCTG-3′; human actin (forward), 5′-CTGGCACCACACCTTCTAC-3′; human actin (reverse), 5′-CATACTCCTGCTTGCTGATC-3′.

*Eco*RI (P5n) and *Bam*HI (P6n) restriction enzyme adapter sites are underlined. Start codons (P5 and P5n) are in bold. Stop codons (P6 and P6n) are in italics.

### Cell lines, RT-PCR analysis and northern blot analysis

The RB and NB cell lines used in this analysis have been previously described [46], [47]. Poly(A)^+^ RNA (1 µg) isolated from chick retina at day 5 of incubation (E5), twelve human RB tumor cell lines, ten human NB tumor cell lines, and total RNA (4 µg) isolated from human fetal retina (8 wks gestation), human fetal brain (8 wks gestation) and human RB tumor biopsies, were reverse-transcribed using an oligo d(T) primer and Superscript reverse transcriptase (Invitrogen). PCR amplification (35 cycles) was carried out using 1/20 of the cDNA generated from these reactions. Primer set P1 and P2 was used for the analysis of the two-exon region deleted in *Dab1-E*; primer set P3 and P4 was used for the analysis of the exon inserted in *Dab1-E*; and primer set P1 and P4 was used for the analysis of both the deletion and insertion regions. Amplification of actin cDNA served as the positive control. DNA fragments were run in 10% polyacrylamide gels in Tris-borate EDTA (TBE) buffer. For sequencing, amplified cDNAs were either sequenced directly or cloned into the pCRII-TOPO TA-overhang cloning vector (Invitrogen) and sequenced.

For amplification of the entire human Dab1-E and Dab1-L open reading frames, a nested primer approach was used. Human fetal retina (8 weeks gestation) cDNA was amplified with the P5/P6 primer set. Amplified cDNAs were purified and re-amplified with the P5n/P6n primer set containing *Eco*RI and *Bam*HI restriction enzyme sites (underlined in *Primers*), respectively. These cDNAs were cloned into pEGFP-C1 (BD Biosciences) and confirmed by sequencing.

For northern blot analysis, two µg of poly(A)^+^ RNA isolated from RB cell lines were electrophoresed in a 6% formaldehyde-1.5% agarose gel in MOPS buffer and transferred to nitrocellulose membrane. The membrane was hybridized with: (a) a 1 kb human *Dab1* 3-end cDNA fragment, and (b) actin cDNA. The signal was visualized by autoradiography.

### Transfection analysis and Reelin treatment

Transfection and confocal microscopy analyses of primary chick retina cultures were performed as previously described [17]. Briefly, primary retinal cultures were prepared from trypsinized E5 chick retinas plated onto glass coverslips (one-twelfth of a retina per 12 mm coverslip). Cells were transfected by calcium phosphate-mediated DNA precipitation and the DNA removed after 16 h. Thirty h later, the cells adhering to coverslips were fixed with 4% paraformaldehyde in phosphate-buffered saline (PBS), permeabilized in 0.5% Triton X-100/PBS and incubated with mouse monoclonal anti-phosphotyrosine antibody (1∶250) (clone PT-66; Sigma) or mouse monoclonal anti-phospho-SFK^Y416^ antibody (1∶50) (clone 9A6; Upstate) followed by Alexa 555-conjugated goat anti-mouse secondary antibody (Invitrogen) (1∶200). The coverslips were mounted on slides using glycerol containing 1 mg/ml *p*-phenylenediamine (PPDA) and 1 µg/ml 4′, 6′-diamidino-2-phenylindole (DAPI) and viewed on a Zeiss LSM 510 confocal microscope.

For Reelin inhibition experiments, Dab1-L-transfected retinal cells were treated with CR-50 antibody (kindly provided by Dr. T. Curran, Children's Hospital of Philadelphia, PA) which interferes with Reelin homopolymerization. After the DNA was removed, 1 µl of CR-50 antibody was added to each well containing 1 ml of medium. Mock treatment was carried out with an equal amount of pre-immune serum. After 24 h, another 1 µl of CR-50 was added for an additional 24 h. Cells were then fixed and stained with anti-phospho-SFK^Y416^ antibody. GFP-positive cells were detected by epifluorescence. Images in Fig. 4 were obtained using the Tile-Scan function of the LSM program. A motorized scanning stage was used in conjunction with the calibrated size of the field of view of each frame. The program automatically moves the specimen to the next field view after each scan and provides a montage of the pre-defined number of scans. Each tile frame size is 1024×1024 pixels. A 3×3 tile was used to construct each image, with a total image size of 3072×3072.

For Reelin treatment of RB522A cells, HEK293T cells were transfected with the pCrl Reelin expression construct (a gift from Dr. T. Curran). The medium was replaced with serum-free OPTI-MEM (Invitrogen) 24 h after transfection. Supernatant was collected 36 h later and concentrated 50X using Amicon Ultra filters (100,000 molecular mass cut-off) (Millipore). RB522A cells were treated with Reelin (1/25) for 30 min.

### Immunofluorescence analysis of human fetal retina sections

Retinal tissue sections were prepared as previously described [17]. Frozen sections were rehydrated in PBS, fixed in 4% PBS-buffered paraformaldehyde and permeabilized in 1% PBS-buffered NP-40. Sections were double-stained with rabbit polyclonal anti-Dab1 antibody prepared against the C-terminus of Dab1 (amino acids 400–555 which are common to both Dab1-E and Dab1-L-like isoforms) (1∶500) (Rockland Immunochemicals) and mouse anti-phospho-SFK^Y416^ antibody (clone 9A6) (1∶25) (Upstate), anti-Islet1 antibody (clone 39.4D5) (1∶500) (Developmental Studies Hybridoma Bank), anti-Ki67 antibody (clone MIB-1) (1∶1000) (Dakocytomation), or anti-AP2α antibody (clone 3B5) (1∶200) (Developmental Studies Hybridoma Bank). The following secondary antibodies were used: Alexa 555-conjugated goat anti-rabbit for Dab1 and Alexa 488-conjugated donkey anti-mouse for SFK^Y416^, Islet1, MIB-1 and AP2α (1∶150) (Invitrogen). The specificity of the anti-Dab1 antibody has been verified by western blot analysis of *Dab1-/-* mouse brain as well as immunostaining of *Dab1-/*- mouse brain tissue. Sections were counterstained with the Hoescht 33258 fluorescent nuclear stain (Invitrogen) and mounted with Fluorosave (Calbiochem). Images were collected with a Zeiss-Axioplan II microscope (Carl Zeiss) equipped with a cooled charge-coupled device camera (Cooke Corporation).

### Western blot analysis, immunoprecipitation and phosphatase treatment

Whole cell lysates were prepared by resuspending cells in 20 mM Tris-HCl, pH 7.5, 150 mM NaCl, 1mM MgCl_2_, 0.1 mM CaCl_2_, 10% glycerol, 0.1% Triton X-100, 1 mM Na_3_VO_4_, 1 mM NaF and 1X Complete (Roche) protease inhibitor cocktail, followed by syringing through a 23G needle. Forty µg protein lysates were electrophoresed through an SDS-polyacrylamide gel. Proteins were transferred to nitrocellulose or PVDF membranes by electroblotting. Blots were immunostained with mouse anti-Reelin (1∶500) (clone 142; Calbiochem), rabbit anti-Dab1 (1∶1000), goat anti-actin (clone I-19; Santa Cruz), rabbit anti-Nova1 (1∶1000; Upstate Cell Signaling Solutions) or goat anti-Nova2 (1∶1500; Santa Cruz Biotechnology) antibodies. Signals were visualized using the ECL or ECL Advance (GE Healthcare) chemiluminescence detection systems.

Immunoprecipitation of Dab1 was carried out with anti-Dab1 antibody (2 µl antibody/500 µg cell lysates) as previously described [22]. For phosphatase treatment, immunoprecipitates bound to protein A Sepharose beads were washed in lysis buffer without phosphatase inhibitors and incubated with lambda phosphatase as previously described [22]. Mouse anti-phosphotyrosine antibody 4G10 (Millipore) was used at a 1∶2000 dilution for western blot analysis.
